# Longitudinal trajectories unravel the complex interplay of medication, cardiovascular events, chronic kidney disease, and mortality

**DOI:** 10.1038/s41598-025-23527-5

**Published:** 2025-10-13

**Authors:** Kaile Chen, Farhad Abtahi, Carlos Fernandez-Llatas, Hong Xu, Fernando Seoane

**Affiliations:** 1 Department of Clinical Science, Intervention and Technology, Karolinska Institutet, Stockholm, 17177 Sweden; 2 Department of Biomedical Engineering and Health System, School of Engineering Sciences in Chemistry, Biotechnology and Health, KTH Royal Institute of Technology, Huddinge, 14157 Sweden; 3 Department of Clinical Physiology, Karolinska University Hospital, Stockholm, 17176 Sweden; 4 SABIEN, ITACA, Universitat Politécnica de Valencia, Valencia, Spain; 5 Division of Clinical Geriatrics, Department of Neurobiology, Care Sciences and Society (NVS), Karolinska Institutet, Stockholm, 17177 Sweden; 6 Department of Medical Technology, Karolinska University Hospital, Stockholm, 17176 Sweden; 7 Department of Textile Technology, University of Borås, Borås, 50190 Sweden

**Keywords:** Cardiology, Diseases, Medical research, Nephrology, Risk factors

## Abstract

Proton pump inhibitors (PPIs) are widely used to treat acid-related disorders, yet concerns persist regarding their association with adverse cardiovascular (CVAE), renal outcomes, and all-cause mortality. We leveraged real-world longitudinal data from the Stockholm CREAtinine Measurements (SCREAM) project to investigate trajectories linking PPI use with chronic kidney disease (CKD), CVAE, and all-cause mortality using a process mining approach. We identified 294,734 new users of PPIs or H2 blockers (H2Bs) with a baseline eGFR ≥ 60 mL/min/1.73 m² and followed them for up to 15 years using a process-mining approach to discover disease trajectories. The association of PPI and CVAE is not significant after accounting for death as a competing event. However, PPI use is significantly associated with a higher subdistribution hazard of CKD and all-cause mortality compared to H2B. Our findings indicate that CKD may act as a mediator in the trajectory *PPI*→*CKD*→*CVAE*, underscore the importance of monitoring renal function in long-term PPI users, and emphasise the need for future prospective trials to clarify cardiovascular risks.

**Supplementary Information:**

The online version contains supplementary material available at 10.1038/s41598-025-23527-5.

## Introduction

Proton pump inhibitors (PPIs), widely prescribed as first-line treatments for conditions such as gastroesophageal reflux disease (GERD) and gastric ulcers, are also commonly used for long-term prophylaxis in clinical practice^1^. However, PPIs have increasingly raised concerns^2^ due to emerging evidence linking their prolonged use to adverse health outcomes, including chronic kidney disease (CKD)^3,4^, cardiovascular adverse events (CVAE)^5,6^, and increased mortality^7^​​.

The association between PPI use and adverse cardiovascular outcomes, including all-cause mortality, remains controversial^8–11^. By contrast, the nephrotoxic potential of PPI is well-established^3,12^. In clinical practice, the relationship between declining renal function and cardiovascular disease is recognised as bidirectional^13^. CKD may increase CVAE risk through mechanisms such as endothelial dysfunction, oxidative stress, and chronic inflammation, while CVAE can accelerate renal decline by impairing renal perfusion and increasing hemodynamic stress^13,14^. This complex interplay makes it challenging to isolate the effects of PPI on disease progression, as CKD and CVAE often coexist and share overlapping risk pathways. While growing evidence^15^ suggests an association between PPI use and CKD progression, the links between PPI, CVAE, and all-cause mortality remain less clear. Given the relatively well-established association between PPI use and CKD, we hypothesised that if PPI use has an impact on CVAE, this effect might be mediated through CKD progression. Specifically, PPI use could contribute to CVAE risk indirectly by promoting the development or worsening of CKD. Randomised clinical trials are often limited by short follow-up periods and may fail to capture such long-term adverse effects, underscoring the need for studies that explore how these chronic conditions evolve over time.

To address this gap, we propose combining process mining methodology with clinical epidemiological real-world data^16^ to visualise and analyse longitudinal transitions between disease events. Process mining is a data-driven approach that uses time-stamped clinical data to automatically generate process maps, illustrating how disease patterns evolve over time. This temporal property makes process mining a promising tool to support causal inference and provide valuable insights into disease dynamics. When applied to large-scale cohort data, this approach can reveal the temporal ordering and interdependencies between PPI use, CKD progression, CVAE, and death.

Our primary aim is to characterise the longitudinal disease trajectories of PPI and H2B users, focusing on the temporal relationships among PPI use, CKD onset or progression, CVAE, and death. Specifically, we aim to (1) assess whether PPI use is associated with increased risk of CVAE, (2) explore, in a real-world setting, whether CKD could act as an intermediate step linking PPI use to CVAE. By integrating process mining with statistical modelling, we seek to provide novel insights into the safety of long-term PPI use and clarify the complex interplay between renal and cardiovascular outcomes.

## Results

### Study population and baseline characteristics

**Fig. 1:**
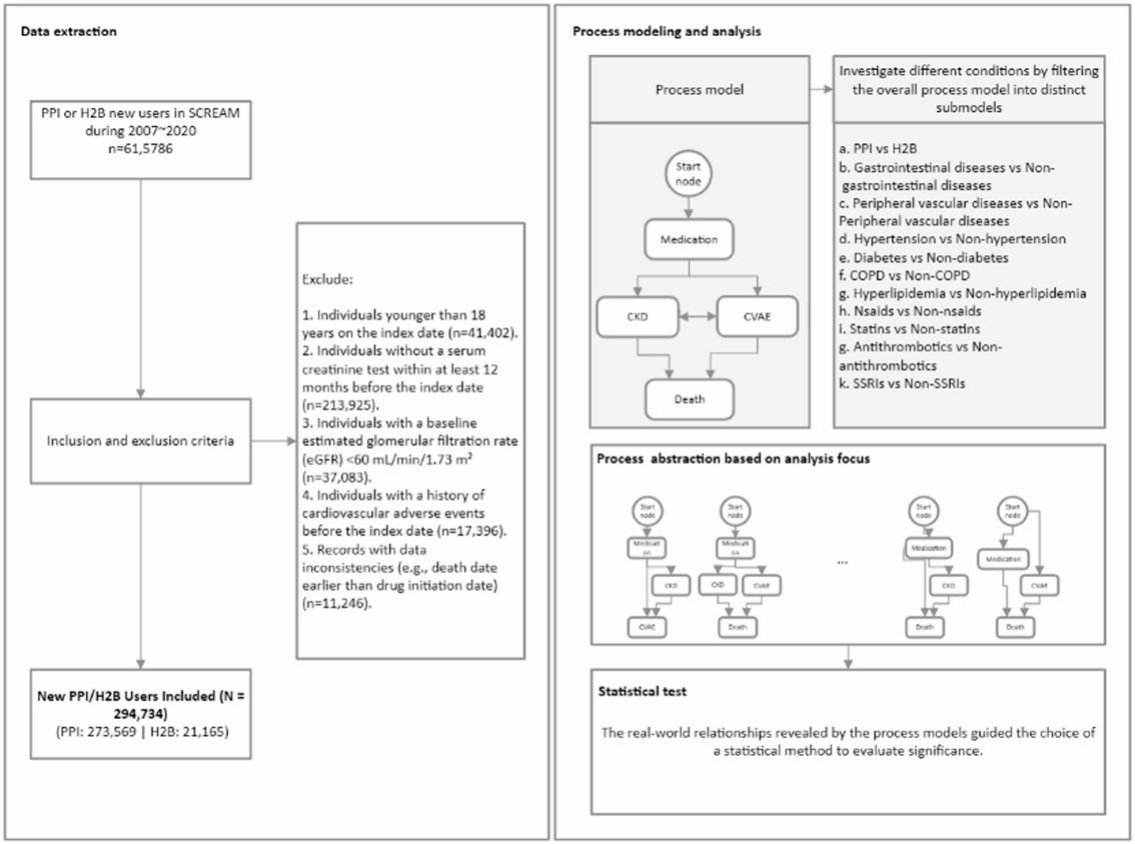
The diagram illustrates the data extraction, process modelling and analysis.This figure includes: A flowchart illustrating the selection of new Proton Pump Inhibitor (PPI) and H2 blocker (H2B) users from the SCREAM database. Process model construction and analysis are performed to explore disease trajectories, with statistical tests subsequently used to assess the patterns identified by the process model.

Between 2007 and 2020, we identified 615,786 new users of PPI or H2B. After applying the inclusion and exclusion criteria (Fig. 1), we included 294,734 individuals in the final cohort: 273,569 PPI users and 21,165 H2B users. Compared to H2B users, PPI users were older, had a higher burden of comorbidities, used more concomitant medications, and exhibited lower baseline kidney function (Supplemental Table 1).

### Process model trajectories

**Fig. 2:**
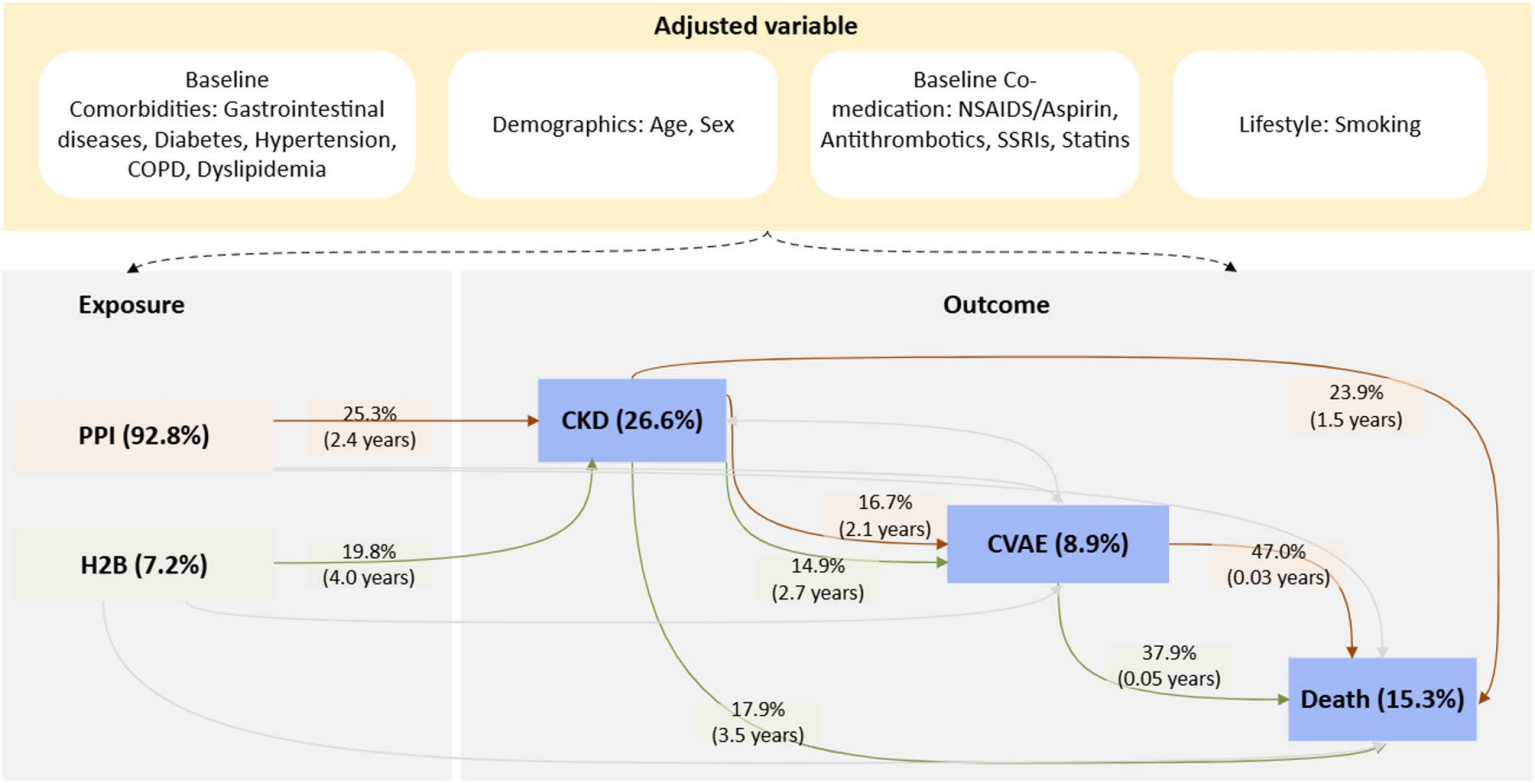
A data-driven DAG (directed acyclic graph) synthesises evidence-based insights with data-driven modelling to represent the disease trajectory and guide the data analysis strategy employed in this study. The node represents the event and displays the relative case frequency (in brackets), which is the proportion of individuals (cases) experiencing the event among all included individuals. The edge (arrow) represents time-ordered sequences of events and displays two statistics, **relative-antecedent frequency (risk)**, which is the proportion of *exposure event* (antecedent) individuals (cases) directly followed by the target individuals (cases); and **median time** (in brackets) represents the time from the exposure event to the outcome or competing event, in years. Only statistically significant transitions between PPI and H2B, with a transition risk greater than 10%, are shown in the indicator.

We developed a process model to represent clinically meaningful sequences of events (Supplemental Fig. 1). The model was abstracted to highlight plausible causal pathways and to generate a corresponding data-driven directed acyclic graph (DAG, Fig. 2), combining both knowledge-driven and data-driven approaches to produce a DAG-like indicator. This abstraction facilitated the identification of key patient trajectories that may represent underlying mechanisms linking exposures to outcomes. Only statistically significant transitions between PPI and H2B with a transition risk exceeding 10% are displayed.

Adjusted variables shown in Fig. 2 were selected based on prior literature and further validated through process mining–based filtering sub-models, which evaluated their associations with both exposure and outcome variables. These sub-models involved generating separate process models for individuals with and without specific conditions (e.g., hypertension) and comparing the distribution of exposures and outcomes between the condition and non-condition groups (Supplemental Fig. 2). All the transitions derived from the process model were then compared between users of PPI and H2B (Table 1).

**Fig. 3:**
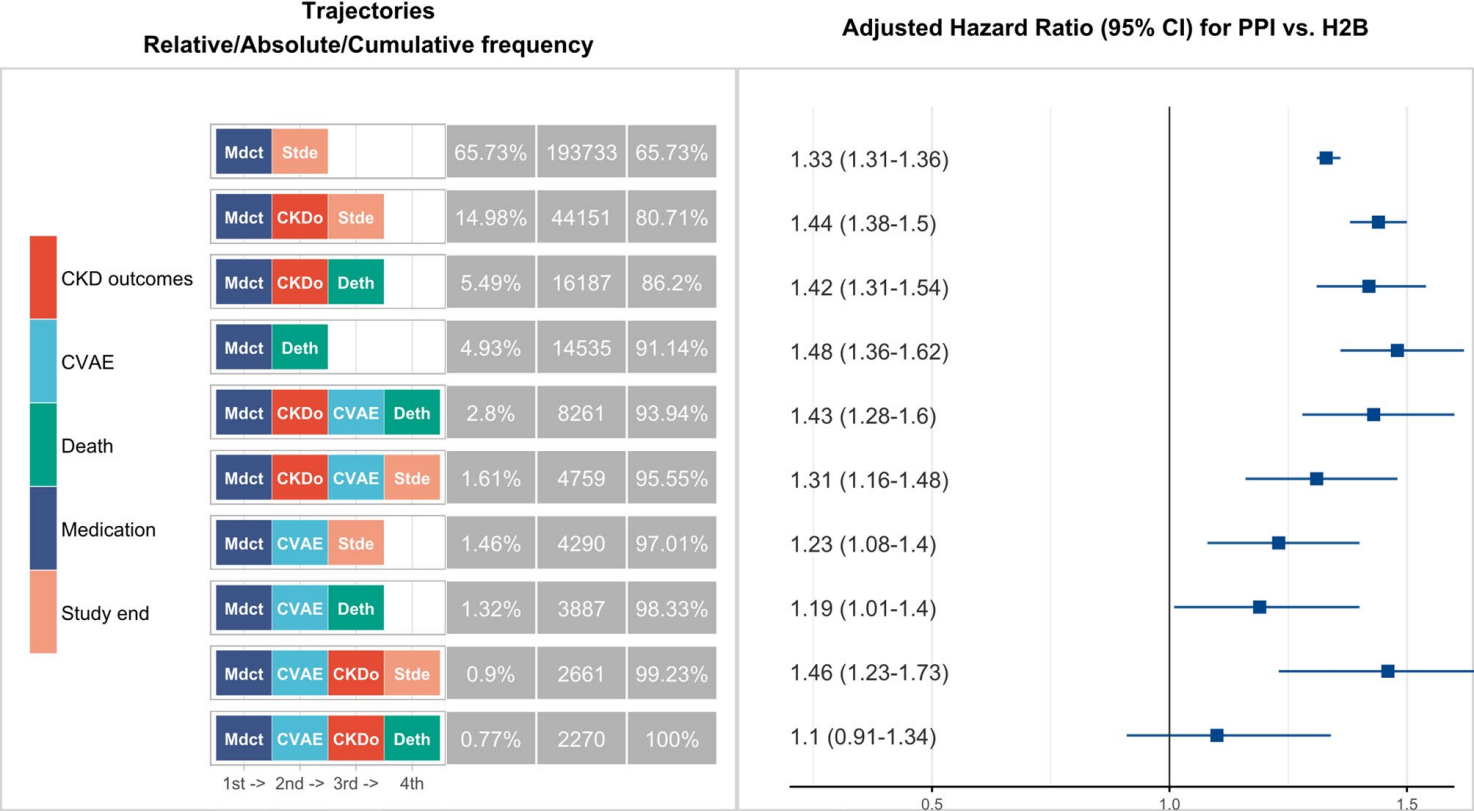
Comparison of trajectories of new users of PPI or H2B, 2006–2021. Trajectories: Chronological disease trajectories including Medication, CKD outcomes, CVAE, Death/Study end; each trajectory is presented with the relative frequency, absolute frequency and cumulative frequency. Trajectories are ordered by frequency. PPI vs. H2B for the following trajectory: hazard ratios and 95% confidence intervals of undergoing a specific trajectory between PPI and H2B users. Estimates were derived from a Cox proportional hazards model with inverse probability weighting (IPW), adjusting for age, sex, eGFR at index, comorbidities, and concomitant medication use at index.

We defined a disease trajectory as the complete sequence of temporal events for each individual. In total, we identified 10 distinct trajectories (Fig. 3). Comparing each trajectory between PPI and H2B users, we found that PPI use was associated with a higher hazard for all trajectories except *Medication → CKD → CVAE → death*, for which the difference between PPI and H2B was not statistically significant.

**Table 1:** Transition event progression rates and rate ratios comparing PPI versus H2B for trajectories derived from the process mining model.

Transitions	PPI				H2B				PPI vs. H2B	
	Events	No. at risk	Risk, %	Median time (IQR), years	Events	No. at risk	Risk, %	Median time (IQR), years	Risk ratio (95% CI)	*p*-value
Key transitions:										
Medication→CKD	69,177	273,569	25.3	2.4 (0.7, 5.7)	4181	21,165	19.8	4.0 (1.4, 7.7)	1.28 (1.24, 1.32)	< 0.001
CKD→CVAE	12,355	73,823	16.7	2.1 (0.4, 4.7)	665	4466	14.9	2.7 (0.6, 5.4)	1.12 (1.05, 1.21)	0.002
CKD→Death	17,659	73,823	23.9	1.1 (0.2, 3.5)	798	4466	17.9	1.4 (0.3 4.3)	1.34 (1.26, 1.43)	< 0.001
CVAE→Death	11,610	24,707	47.0	0.03 (0, 1.2)	538	1421	37.9	0.05 (0, 1.8)	1.24 (1.16, 1.33)	< 0.001
Other observed transitions in the process model:										
Medication→CVAE	12,352	273,569	4.5	2.9 (1.0, 5.9)	756	21,165	3.6	3.9 (1.6, 7.3)	1.26(1.18, 1.36)	< 0.001
Medication→Death	13,967	273,569	5.1	1.5 (0.4, 4.1)	568	21,165	2.7	3.5 (1.3, 6.7)	1.90 (1.75, 2.07)	< 0.001
CVAE→CKD	4646	24,707	19.0	0.7 (0.1, 2.4)	285	1421	20.1	1.4 (0, 1.8)	0.94 (0.84, 1.04)	0.238

**Risk is defined as the proportion of individuals with the outcome among those at risk, calculated as *Events ÷ No. at risk*. **Risk ratio (RR)**: The crude RR compares risks between groups (PPI vs. H2B, or CKD vs. non-CKD) as **Risk₍exposed₎ ÷ Risk₍reference₎**, with 95% CIs derived using the log method. Median time (Interquartile Range, IQR) represents the time from the exposure event to the outcome or competing event, in years.

### Observed death as a competing risk

**Fig. 4:**
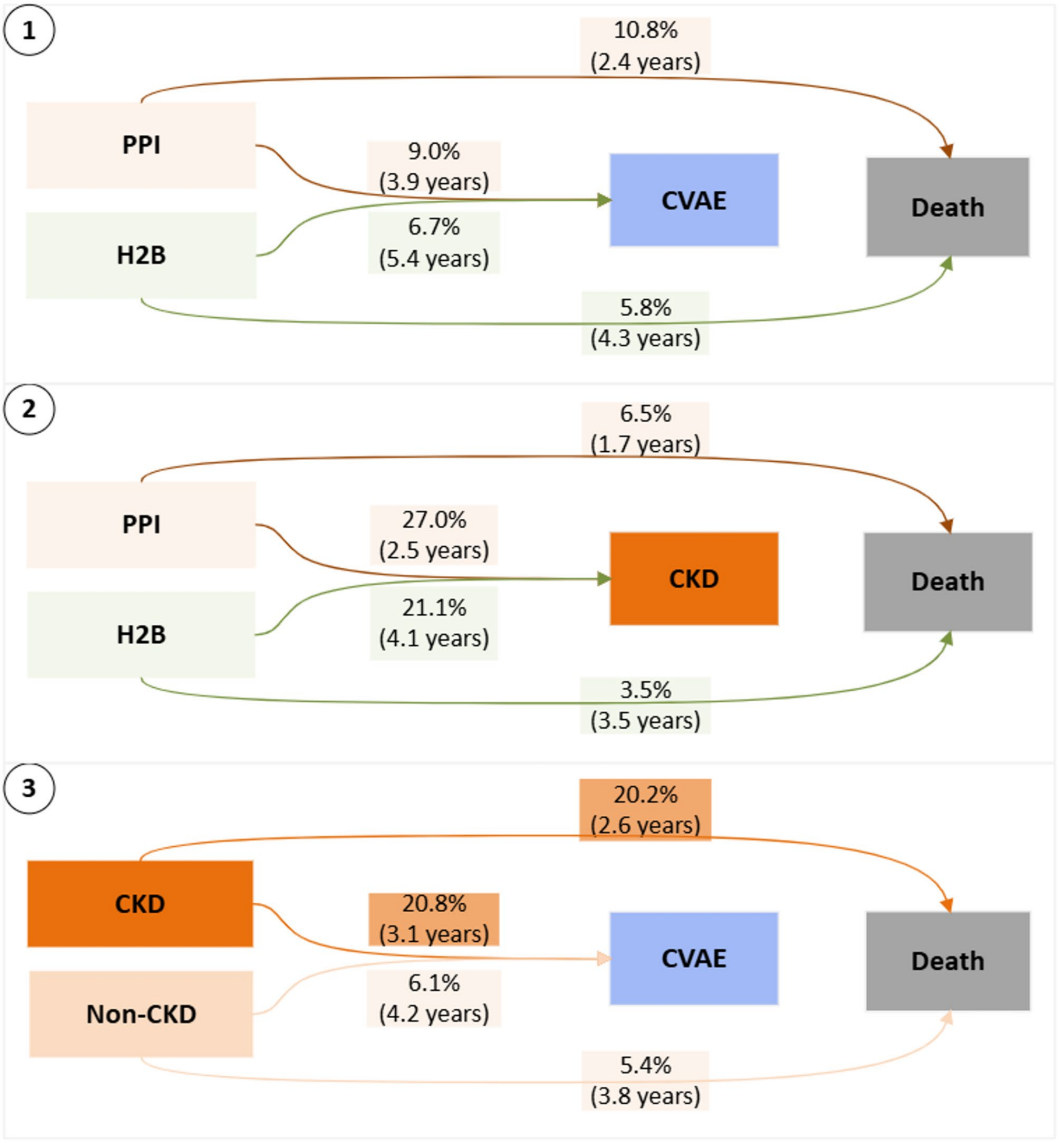
Data-driven DAGs depict the influence of death as a competing risk when evaluating CVAE and CKD outcomes. The node represents the event. The edge (arrow) represents time-ordered sequences of events and displays two statistics, relative-antecedent frequency, which is the proportion of source (antecedent) cases directly followed by the target cases; and median duration (in brackets) from antecedent event to the target event.

Mortality differed by exposure groups (PPI vs. H2B users) and may prevent subsequent occurrence or observation of CVAE or CKD, thus indicating death as a competing risk (Fig. 4). We applied process abstraction by analysing each outcome separately while treating death as a competing event and excluding transitions related to the other outcome. When the main outcome is CVAE (with death as the competing event and ignoring CKD transitions), the death risk was significantly higher among PPI users (10.8%) compared to H2B users (5.8%) (Fig. 4. *No.1*). Similarly, when CKD was the primary outcome (death as the competing event, ignoring CVAE transitions), PPI users again had a higher death risk (6.5%) compared to H2B users (3.5%) (Fig. 4. *No.2*). In a landmark analysis starting two years after the index date, we examined CKD as an exposure for subsequent CVAE risk. Here too, death served as a competing event: 20% of CKD cases resulted in death, compared to 5% in the Non-CKD group (Fig. 4. *No.3*). These differences indicate that death is associated with exposure and must be handled as a competing event.

Consequently, we used the Fine–Gray competing risk model to estimate adjusted subdistribution hazard ratios (SHRs). As shown in Table 2, the association between PPI use and CVAE was not statistically significant after accounting for death and adjusting for baseline confounders. However, PPI use significantly increased the hazard for CKD. Likewise, CKD onset significantly increased the hazard for CVAE. The specifics of competing risk models are detailed in Supplemental Table 2.

**Table 2:** Risks, crude risk ratios (RR), and adjusted subdistribution hazard ratios (SHR) for key disease trajectories by exposure group.

Exposure: medication (PPI/H2B)	PPI				H2B				PPI vs. H2B		
	Events	No. at risk	Risk, %	Median time (IQR), years	Events	No. at risk	Risk, %	Median time (IQR), years	Risk ratio (95% CI)	Adjusted SHR, 95%CI	*P*
Focused outcome: CVAE											
Medication→CVAE	24,707	273,569	9.0	3.9 (1.5, 7.3)	1421	21,165	6.7	5.4 (2.4, 8.8)	1.35 (1.28, 1.42)	0.97 (0.92, 1.02)	0.307
Competing trajectory: Medication→Death	29,476	273,569	10.8	2.4 (0.7, 5.8)	1246	21,165	5.8	4.3 (1.6, 8.1)	1.83 (1.73, 1.93)	NA	NA
Focused outcome: CKD											
Medication→CKD	73,823	273,569	27.0	2.5 (0.7, 5.8)	4466	21,165	21.1	4.1 (1.5, 7.8)	1.28 (1.25, 1.31)	1.10 (1.07, 1.13)	< 0.001
Competing trajectory: Medication→Death	17,679	273,569	6.5	1.7 (0.5, 4.5)	743	21,165	3.5	3.5 (1.3, 7.0)	1.84 (1.71, 1.98)	NA	NA

Exposure: CKD (yes/no)	CKD				Non-CKD				CKD vs. Non-CKD		
	Events	No. at risk	Risk, %	Median time (IQR), years	Events	No. at risk	Risk, %	Median time (IQR), years	Crude RR, 95% CI	Adjusted SHR, 95%CI	p
Focused outcome: CVAE											
CKD→CVAE	4680	23,311	20.1	3.1 (1.3, 5.8)	13,578	225,970	6.0	4.2 (1.9, 7.0)	3.34 (3.24, 3.44)	1.34 (1.29, 1.39)	< 0.001
Competing trajectory: CKD→Death	4709	23,311	20.2	2.6 (1.0, 5.2)	12,155	225,970	5.4	3.8 (3.1, 9.7)	3.76 (3.64, 3.87)	NA	NA

*Risk is defined as the proportion of individuals with the outcome among those at risk, calculated as *Events ÷ No. at risk*. **Risk ratio (RR)**: The crude RR compares risks between groups (PPI vs. H2B, or CKD vs. non-CKD) as **Risk₍exposed₎ ÷ Risk₍reference₎**, with 95% CIs derived using the log method. Median time (Interquartile Range, IQR) represents the time from the exposure event to the outcome or competing event, in years. **Adjusted SHR**s are estimated using a multivariable Fine–Gray subdistribution hazards model accounting for all-cause mortality as a competing risk and adjusting for baseline covariables (95% CIs reported). Abbreviations: SHR, subdistribution hazard ratio; CI, confidence interval; CKD, chronic kidney disease; CVAE, cardiovascular adverse events; H2B, H₂ blocker; PPI, proton pump inhibitor.

### The potential mediation effect of CKD

**Fig. 5:**
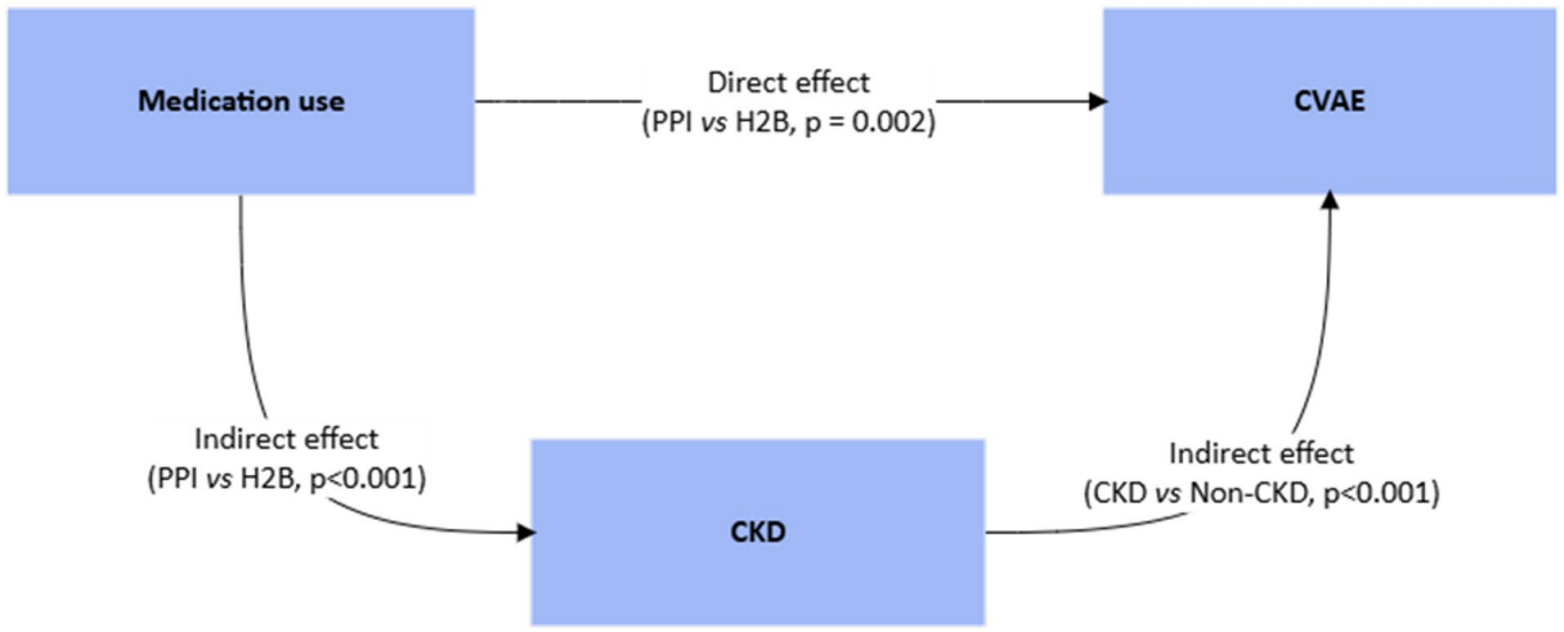
A data-driven DAG illustrates the mediation framework. The **first indirect effect** (medication use → CKD) is represented by the association between medication exposure (PPI vs. H2B) and CKD, with the p-value derived from a competing risk model for CKD as the outcome. The **second indirect effect (**CKD → CVAE**)** is represented by the association between CKD status (CKD vs. non-CKD) and CVAE, with the p-value obtained from a competing risk model for CVAE as the outcome. The **direct effect** is represented by the association between medication exposure (PPI vs. H2B) and CVAE with adjusting for CKD status in the competing risk model.

The process model indicated temporal interplay between CKD and CVAE, with CKD frequently preceding CVAE. This temporal pattern suggests kidney function impairment may serve as an intermediate pathway linking PPI exposure to CVAE. To explore this pathway, we abstracted a process indicator including three key events: medication exposure, CKD, and CVAE (Fig. 5). In traditional mediation analysis, the total effect of an exposure on an outcome can be decomposed into a direct effect (medication use → CVAE) and an indirect effect (medication use → CKD → CVAE). Although the total effect of PPI use on CVAE was not statistically significant in the competing risk model (Table 2), PPI use was significantly associated with an increased risk of CKD, and CKD in turn was significantly associated with a higher hazard of CVAE. This pattern suggests a possible indirect effect of PPI use on CVAE mediated through CKD.

## Discussion

In this cohort study, we examined the longitudinal trajectories of PPI or H2B use in relation to CKD, CVAE, and all-cause mortality. Using a process-mining approach, we discovered event sequences over time and found that PPI users experienced faster progression along these trajectories compared with H2B users. After accounting for death as a competing risk and adjusting for baseline covariates, the subdistribution hazard of CVAE was not significantly different between PPI and H2B users. However, the transition speed from medication use to CKD, and from CKD to subsequent CVAE, remained significantly higher among PPI users after adjusting for death as a competing event.

### Insights from the process model

The process model was constructed from time-stamped clinical events, producing a time-ordered sequence that enables a data-driven exploration of potential causal relationships. The process model reveals that the PPI group exhibits both a higher proportion of patients and a shorter median progression time along all disease trajectories. Furthermore, mortality is distributed differently between the PPI and H2B groups, with a substantial proportion of deaths occurring before the onset of CKD or CVAE. This pattern suggests that death acts as a competing risk, which must be accounted for when assessing the association between PPI use and CKD or cardiovascular events.

Additionally, we observed that CKD frequently precedes CVAE from the process model, suggesting that CKD may serve as a mediator in the pathway linking PPI use to cardiovascular adverse events. A smaller proportion of patients developed CVAE before CKD. According to collider theory^17^, when two factors both cause a third factor (e.g., *Medication → CKD ← CVAE*), that third factor can act as a collider. In this context, CKD could theoretically function as a collider between medication use and CVAE. However, we found no significant difference between the PPI and H2B groups in the transition from CVAE to CKD. This pattern suggests that, in the current population, CKD is more likely to function as a mediator rather than a collider. To further explore this, we evaluated the associations for the transitions *Medication → CKD*, *CKD → CVAE*, and the full trajectory *Medication → CKD → CVAE → Death/End*. All of these, whether partial transitions or the complete trajectory, showed significantly higher hazards in PPI users compared with H2B users. This indirect transition, *PPI → CKD → CVAE*, supports the hypothesis that CKD mediates the relationship between PPI use and cardiovascular adverse events. Although the total effect of PPI use on CVAE risk was not statistically significant, the indirect pathway remained clinically and mechanistically meaningful. We then discussed several explanations for the lack of a significant total effect. First, in real-world settings, complex interactions may create opposing causal pathways that counterbalance potential adverse effects. Second, death may act as a competing risk, masking cardiovascular outcomes. Third, PPIs have protective effects on the gastrointestinal tract, particularly when co-administered with drugs that increase bleeding risk, and may offset some cardiovascular risks. Therefore, even in the absence of a significant overall association, investigating specific mechanistic pathways remains valuable.

### The association of PPI with CVAE and clinical implications

The total effect of PPI use on CVAE risk was not statistically significant after adjusting for potential confounders and accounting for death as a competing event. In our cohort, all-cause mortality was significantly higher in the PPI group, which might reflect their worse overall health profile. Death represents a competing event for CVAE, meaning that many high-risk individuals in the PPI group may have died from other causes before developing a CVAE.

When applying the Fine–Gray subdistribution hazards model, which accounts for death as a competing risk, the estimated cumulative incidence of CVAE was lower than that obtained from the Cox proportional hazards model. This difference arises because the Fine–Gray model recognises that individuals who die before experiencing a CVAE are no longer at risk for the event and thus are excluded from the risk set in subsequent follow-up. By contrast, the Cox hazards regression model treats such deaths as non-informative censoring, implicitly assuming that censored individuals would have the same future risk as those who remain alive. In populations with high competing mortality, this can lead to overestimation of the cumulative event probability. The difference between the Cox and Fine-Gray becomes clearer when the competing event is common and occurs early during follow-up, as death often happens in older or high-risk patient populations^18,19^. This explains why, when using the Cox model, certain trajectories between PPI and H2B users (e.g., *Medication → CVAE → Death/End*) appeared statistically significant, likely because the PPI group had shorter follow-up and faster progression across all trajectories. However, when applying the competing risk model to account for death, the association of PPI on CVAE were no longer statistically significant.

In clinical practice, PPIs are often prescribed prophylactically for patients receiving NSAIDs or antiplatelet therapy who are at higher risk of upper gastrointestinal bleeding^20^. In such cases, the cardiovascular benefits of antiplatelet therapy may outweigh potential harms. From a biological perspective, although PPI use has been linked to possible cardiovascular risks^21,22^ by potentially diminishing clopidogrel’s benefits through reduced bioavailability of its active metabolites via competitive inhibition of cytochrome P450 isoenzyme 2C19 (CYP2C19), which is critical for clopidogrel activation, PPIs may also offer protective effects. These include anti-inflammatory properties^23^ and prevention of gastrointestinal bleeding, thereby allowing uninterrupted use of cardioprotective agents such as antiplatelets and anticoagulants^24^. Furthermore, clinicians often choose PPIs over H2Bs for patients with more severe gastrointestinal disease^20^. Through closer follow-up or more intensive clinical management, these PPI users may have had their cardiovascular risk mitigated, potentially reducing the incidence of CVAE.

These methodological, clinical, and pharmacological factors may partly explain why our study did not detect a significant overall association between PPI use and CVAE risk. This interpretation is consistent with findings from previous research. For example, a meta-analysis reported no significant association between PPI use and cardiovascular events but found an increased risk of cardiovascular mortality^8^. Higher cardiovascular risk with PPI use is often reported in studies comparing PPI users to non-users^25^. In contrast, our study compared PPI users with H2B users in a new-user, active-comparator design, which helps reduce indication bias because prevalent PPI use may reflect prior cardiovascular conditions. Moreover, clinical trials have generally shown that PPI use does not increase cardiovascular risk when combined with antiplatelet therapy^22,26^.

However, when a patient has CKD, the situation may be different. As discussed earlier, the indirect effect of PPI use on CVAE through CKD may still be present, even if the total effect is not statistically significant. On one hand, our findings indicate that PPI use is associated with an increased hazard of developing CKD, consistent with previous concerns about the potential nephrotoxic effects of PPIs^15,16,27^. On the other hand, CKD progression can amplify cardiovascular risk through mechanisms such as endothelial dysfunction and systemic inflammation^28^, while CVAE can further worsen renal function through hemodynamic stress^29^. In clinical practice, healthcare providers are usually very cautious when prescribing medications to patients with CKD. For example, long-term NSAID use, known to be nephrotoxic, is generally avoided, and some cardioprotective agents are underused even though they show benefits for cardiovascular disease and CKD, such as Sodium-glucose cotransporter-2 inhibitors (SGLT2i)^30^ and glucagon-like peptide-1 receptor agonists (GLP-1RA)^31^. As a result, cardiovascular risk may increase both because of the underlying CKD and the reduced use of protective medications. Therefore, clinicians should be particularly cautious when prescribing PPIs to CKD patients and ensure regular monitoring of renal function during long-term therapy.

### Comparison of the process mining approach with related work

Several previous studies have analysed temporal disease trajectories using registry or electronic health record data. Jensen et al.^32^ identified statistically significant, temporally ordered disease pairs and linked them into fixed-length trajectories, while Giannoula et al.^33^ applied dynamic time warping to cluster trajectories with similar temporal shapes. Haug et al.^34^ modelled multimorbidity patterns leading to cardiovascular mortality, and Paik et al.^35^ mapped mortality-centred disease sequences. More recent developments include Lion et al.^36^, who proposed an interval-based DTW method for similarity matching, and Herzeel et al.^37^, who developed the Patient Trajectory Analysis Library to extract and cluster relevant trajectories. Our approach shares similarities with prior work on temporal disease trajectory analysis, which all aim to identify meaningful temporal patterns in patient event histories. Like these methods, process mining uses longitudinal data to reveal temporal ordering among events and can support exploratory analyses for hypothesis generation. However, process mining offers several advantages. First, it reconstructs complete patient journeys of varying lengths, preserving the full sequence of events for each individual (from initial exposure through intermediate diagnoses and outcomes) without restricting the analysis to a fixed number of steps. Second, it integrates heterogeneous event types, including diagnoses, treatments, laboratory results, and mortality, into a single model. Finally, its feasibility and utility in large-scale epidemiological data have been validated in our previous work tracing eGFR trajectories^27^ and proposing a framework^16^ to combine process mining with epidemiological study designs, providing a strong methodological foundation for its application in the present study.

### Strengths, limitations and implications for future research

A key strength of this study is the use of a process mining methodology in a large real-world cohort, enabling the visualisation of how multiple disease events evolve over time. This approach provides an intuitive understanding of disease progression, particularly when multiple comorbidities and events, such as CKD and CVAE, are intertwined. We applied a new user design together with an active comparator design^38^, using a one-year washout period prior to drug initiation to ensure the inclusion of only new PPI or H2B users. This design minimises immortal time bias compared with the prevalent user design. Furthermore, using H2B as an active comparator helps reduce indication bias, as both drugs share similar clinical indications (e.g., gastrointestinal disease). Although PPI users had poorer baseline health at index, this design still offers a more balanced comparison than using non-PPI users as the control group.

Nonetheless, our study’s observational design may be affected by residual confounding despite using a new user design and active comparator and adjusting for potential confounders, and unmeasured factors such as diet or socioeconomic status could influence the results. Besides, as exposure was defined at baseline using the dispensation date of the first PPI or H2B prescription, we could not account for subsequent discontinuation or switching, which may have led to some degree of exposure misclassification. We nevertheless chose an intention-to-treat (ITT) approach over an as-treated design to avoid bias from treatment changes that may be influenced by early symptoms or evolving health status (e.g., informative censoring). This approach is widely used in pharmacoepidemiology as it preserves comparability between exposure groups and maintains the original cohort structure.

The Swedish national health registries capture information generated in daily clinical practice across the entire population, providing one of the most comprehensive and reliable sources for longitudinal epidemiological research. As such, our study benefits from high-quality, population-wide coverage. Nevertheless, certain clinically relevant variables are not included due to legal and ethical restrictions, such as ethnicity. Although ethnicity data are not available by law, the potential misclassification of eGFR is minimal, as the vast majority of residents in the Stockholm region are of Caucasian origin. Finally, because SCREAM includes only individuals with at least one serum creatinine measurement, our cohort represents people who have interacted with the healthcare system, which may overrepresent individuals with comorbidities. However, this population is particularly relevant for our research question, as it reflects the group in whom CKD is most likely to be detected and monitored in clinical practice.

## Conclusion

This study provides new insights into the risks and disease trajectories associated with PPI use. While PPI users had more health problems, higher risks of CKD and mortality, no significant difference in the hazard of CVAE was observed compared with H2B users in the competing risk model. The real-world trajectory analysis suggests that a potential mediation pathway may play an important role in disease progression, in which PPI use could increase the risk of CVAE indirectly through CKD. However, this mediation hypothesis, derived from temporal disease trajectories, needs further validation. Given the widespread use of PPIs, future prospective studies or randomised controlled trials are needed to clarify the cardiovascular effects of long-term PPI therapy, particularly among patients with, or at high risk for, CKD.

## Methods

### Study design and settings

This is a retrospective cohort study that uses an active comparator and new-user design^38^. We used data from the Stockholm CREAtinine Measurements (SCREAM) project, a comprehensive healthcare utilisation cohort covering the Stockholm region, Sweden. SCREAM includes healthcare data from all residents who underwent creatinine measurements (2006–2021), with detailed information on demographics, healthcare visits, hospitalisations, and laboratory measurements^39^. The current research period is from January 1st, 2006, to December 31st, 2021. Medication use was identified through pharmacy dispensations recorded using Anatomical Therapeutic Classification (ATC) codes, linked via the Swedish Prescribed Drug Registry. This study was conducted in compliance with the Declaration of Helsinki. It was approved by the Swedish National Board of Welfare and the Stockholm Regional Ethics Review Board, which waived the requirement for informed consent due to the retrospective nature of the study and the use of de-identified registry data.

### Participants

A cohort of new users of PPI or H2B from SCREAM with eGFR > = 60 mL/min/1.73 m^2^ and without a history of hospitalised cardiovascular adverse events. The index date was defined as the first dispensation date of PPI or H2B between 2007 and 2020. Here, we used a new drug user design and an active comparator design to minimise immortal time bias and confounding by indication^38^. In line with a new-user design, the year 2006 acted as a washout period to exclude prevalent users, ensuring that only individuals with a first dispensing of PPI or H2B between January 1, 2007, and December 31, 2020, were included. The year 2021 was used to ensure that all subjects had at least 1 year of follow-up time. In the comparator design, H2B was chosen as an active comparator for PPI to minimise indication bias, as both drugs share similar clinical indications. The ATC codes used for identifying PPI and H2B are shown in Supplemental Table 3. We followed up individuals from the PPI/H2B dispensation date until the death, emigration or the end date of the study (December 31, 2021). We applied an intention-to-treat (ITT) approach, assigning exposure status at the index date based on the first dispensation of PPI or H2B recorded in the national prescribed drug register, and following participants for the entire study period regardless of subsequent discontinuation or switching.

Individuals were excluded if (a) the estimated glomerular filtration rate (eGFR) < 60 mL/min/1.73 m^2^ before the index date (The calculation of eGFR used the Chronic Kidney Disease Epidemiology Collaboration (CKD-EPI) 2009 creatinine equation^40^); (b) the hospitalisation history of cardiovascular adverse events before the index date; and (c) age < 18 years old.

### Variables

#### Outcome events

We applied principles from process mining theory to explore the longitudinal progression of outcome events. The process begins at the point of medication dispensation (index date), and events of interest were captured throughout the study period. Thus, we can visualise and analyse their sequence over time in a process model. The primary events of interest are summarised in Table 3.

**Table 3:** Description and definition of outcome events in this cohort study.

Event	Event Description	Definition
Index date	Dispensation date of PPI or H2B	Cohort entry begins at the index date
CKD Outcomes Adverse outcomes related to chronic kidney disease	eGFR Decline	≥ 30% decline from the index eGFR
	CKD Incidence	eGFR < 60 mL/min/1.73 m²
	Kidney Replacement Therapy (KRT)	Dialysis or kidney transplantation, confirmed by the Swedish Renal Registry
	Death due to CKD	Classified under ICD-10 code N18
Cardiovascular Adverse Events (CVAE) Adverse cardiovascular events	Myocardial Infarction	Identified using ICD-10 codes recorded during inpatient visits: I21, I22
	Heart Failure	Identified using ICD-10 codes recorded during inpatient visits: I099, I110, I130, I132, I255, I420, I425, I426, I527, I428, I429, I43, I50, P290
	Stroke	Identified using ICD-10 codes recorded during either inpatient visits: I60, I61, I63, I64
	Death due to Cardiovascular Events	Death attributed to cardiovascular causes defined by ICD-10 codes I21, I22, I099, I110, I130, I132, I255, I420, I425, I426, I527, I428, I429, I43, I50, P290, I60, I61, I63, I64
Death	All-cause mortality, death from any cause	Recorded at any point during follow-up
Study end	The date on which follow-up ended for participants who did not experience any events.	Follow-up ended on December 31, 2021, or on the date of emigration from Sweden, whichever occurred first

#### Covariates

Covariates were selected based on associations or clinical relevance to both the outcomes and the exposure. These covariates were assessed as of the index date, meaning that covariates were included in the analysis only if they had recorded data on or before the index date. The covariates included age, sex, kidney function (using eGFR), comorbidities, concomitant medications, and smoking status.

Comorbidities with indications for acid-suppression therapy, such as gastroesophageal reflux disease, Barrett’s oesophagus, ulcer disease, Helicobacter pylori infection, and upper gastrointestinal tract bleeding, were extracted. Additional comorbidities included hypertension and diabetes mellitus, both supplemented by records of medications for hypertension and diabetes treatment, as well as peripheral vascular disease, chronic obstructive pulmonary disease, and dyslipidaemia. Concomitant medications included nonsteroidal anti-inflammatory drugs (NSAIDs)/aspirin, statins, antithrombotic AND selective serotonin reuptake inhibitors (SSRIs). A summary of ICD-10 (International Statistical Classification of Diseases and Related Health Problems, 10th Revision) codes for all included diseases, along with ATC codes for the exposure and concomitant medications, is provided in Supplemental Table 3.

### Statistical methods

This study applied a process mining methodology adapted for observational epidemiology studies^16^. We used the process discovery technique to model the progression of diseases, tracing the trajectory from medication initiation to each relevant clinical event up to the end date of the study. The *BupaR*^41^ package in R was employed to conduct process discovery and generate a process model to visualise and analyse the progression of events across the cohort. We then applied process abstraction^16^ to convert the original process model into a set of data-driven Directed Acyclic Graph (DAG), each targeting specific events, to facilitate a more structured and focused analysis. The process model visualises disease trajectories over time using a direct-follows graph. In this representation, each event is depicted as a node, while arrows connecting two events signify that one event directly follows the other in a chronological sequence.

We defined a full **trajectory** (i.e. a trace variant) as the complete sequence of clinical events from the date of medication initiation to the end of follow-up or death. The trace variants were identified using process discovery by sorting events chronologically for each individual by their timestamps, creating a complete “trace” of activities from start to finish (e.g., Medication → CKD outcomes → CVAE → Death). We then grouped all individuals with the same sequence of activities into a trace variant. In other words, each trace variant represents a unique pattern of events. Hazard ratios (HRs) and 95% confidence intervals (CIs) were used to compare trajectories between PPI and H2B groups, incorporating inverse probability weighting (PSW) to adjust for age, sex, baseline eGFR, comorbidities, and other medication use.

A **transition** was defined as a direct change from one clinical event (antecedent) to another (consequent) within any trajectory, regardless of the preceding or subsequent events. The transitions were compared using risk, calculated as the proportion of individuals who experienced the consequent event among those at risk at the antecedent event, and risk ratio (RR), calculated as the ratio of the risk in the exposure group (e.g. PPI) to that in the reference group (e.g. H2B). The RR was reported with its corresponding 95% confidence interval (CI), estimated using the Wald method. We also computed durations (i.e., years between events) to gain insight into the typical timespans of each transition.

Baseline characteristics were first compared between the new-user cohorts of PPI and H2B. Subsequently, we filtered the process model into sub-models for individuals with and without specific conditions (e.g., hypertension) and compared the distributions of exposures and outcomes between these groups. Conditions associated with both the exposure and the outcome were considered potential confounders and were adjusted for in subsequent statistical models. Continuous variables with normal distributions were summarised using means and standard deviations, while those with non-normal distributions were reported as medians with interquartile ranges (IQRs). Categorical variables were summarised by absolute frequencies and proportions.

The statistical model was selected based on insights from the process map. A Fine-Gray competing risk model^42^ was employed to assess the association between exposure and outcomes. The *cmprsk*^43^ package in R was used for competing risk modelling. We used this approach to estimate the association between exposure and outcome while adjusting for potential confounders (co-variables adjusted for are listed in Supplemental Table 2) and accounting for the competing risk of all-cause death. We performed a landmark analysis to assess the association between CKD and CVAE. Based on our data, the median time to CKD development among all included individuals was approximately two years after the index date; therefore, we set the landmark time at two years post-index. At this landmark, individuals who had developed CKD were classified as the exposed group, and those without CKD were classified as the comparator group. Subsequent incidence of CVAE was then compared between the two groups.

All statistical analyses and data visualisations were performed using R version 4.4.3.

## Supplementary Information

Below is the link to the electronic supplementary material.

Supplementary Material 1

## Data Availability

The SCREAM contains sensitive personal data that cannot be publicly shared due to GDPR regulations. We welcome collaboration project proposals that adhere to GDPR, national, and institutional regulations concerning data sharing and access. For inquiries, please contact Prof. Juan-Jesus Carrero [juan.jesus.carrero@ki.se](mailto: juan.jesus.carrero@ki.se) .
