# Supplementary material for: Longitudinal trajectories unravel the complex interplay of medication, cardiovascular events, chronic kidney disease, and mortality
Source: Sci Rep. 2025 Oct 13;15:35577. doi: 10.1038/s41598-025-23527-5 (PMC12518710; doi:10.1038/s41598-025-23527-5)
Supplement: Supplementary file 1 — Supplementary Material 1 [file 41598_2025_23527_MOESM1_ESM.docx]

Supplemental tables and figures

Contents

[Supplemental Table 1. 2](#_Toc210210692)

[Supplemental Figure 1. 3](#_Toc210210693)

[Supplemental Table 2. 4](#_Toc210210694)

[Supplemental Table 3. 7](#_Toc210210695)

[Supplemental Figure 2.. 12](#_Toc210210696)

### Supplemental Table 1. Baseline characteristics of new users of PPI and H2B from SCREAM (2006-2021)

|  | Overall | | Hospitalisation | |
| --- | --- | --- | --- | --- |
|  | PPI, n=269298 | H2b, n=21323 | PPI, n=273569 | H2b, n=221165 |
| Age, median (IQR) | 52 (38,65) | 43 (30,58) | 54 (40,67) | 44 (31,60) |
| Age stratifications |  |  |  |  |
| 19~45 | 98571 (36.6) | 11233 (52.7) | 92119 (33.7) | 10639 (50.3) |
| 46~64 | 96835 (36) | 6275 (29.4) | 98111 (35.9) | 6373 (30.1) |
| 65~80 | 60182 (22.3) | 2931 (13.7) | 66375 (24.3) | 3183 (15) |
| 81~ | 12111 (4.5) | 493 (2.3) | 15413 (5.6) | 586 (2.8) |
| Baseline eGFR (mL/min/1.73m^2^), median (IQR) | 95.5 (83.4,108.5) | 101.1 (87.7,114.9) | 94.4 (82.4,107.3) | 100.3 (86.8,114.2) |
| Sex n**%** |  |  |  |  |
| Female | 158328 (59) | 13427 (63) | 158188 (58) | 13251 (63) |
| Male | 110970 (41) | 7896 (37) | 115381 (42) | 7914 (37) |
| Comorbidity**,** n**%** |  |  |  |  |
| Gastrointestinal diseases | 27155 (10.1) | 1325 (6.2) | 28480 (10.4) | 1350 (6.4) |
| Peripheral vascular disease | 4053 (1.5) | 161 (0.8) | 5354 (2) | 218 (1) |
| Hypertension | 101879 (37.8) | 5468 (25.6) | 110451 (40.4) | 5843 (27.6) |
| Diabetes mellitus | 25231 (9.4) | 1396 (6.5) | 27705 (10.1) | 1475 (7) |
| Chronic obstructive pulmonary disease (COPD) | 35547 (13.2) | 2376 (11.1) | 37047 (13.5) | 2405 (11.4) |
| Dyslipidemia | 13884 (5.2) | 634 (3) | 18367 (6.7) | 814 (3.8) |
| Concomitant medication, n% |  |  |  |  |
| NSAIDs, aspirin | 179801 (66.8) | 11785 (55.3) | 179896 (65.8) | 11660 (55.1) |
| Statins | 42260 (15.7) | 2093 (9.8) | 48812 (17.8) | 2398 (11.3) |
| Antithrombotics | 66864 (24.8) | 2625 (12.3) | 75322 (27.5) | 3004 (14.2) |
| SSRIs | 51798 (19.2) | 3700 (17.4) | 52002 (19) | 3628 (17.1) |
| Smoking, n**%** | 2203 (0.8) | 113 (0.5) | 2524 (0.9) | 127 (0.6) |

### Supplemental Figure 1.Process models for PPI or H2B new users on disease trajectory


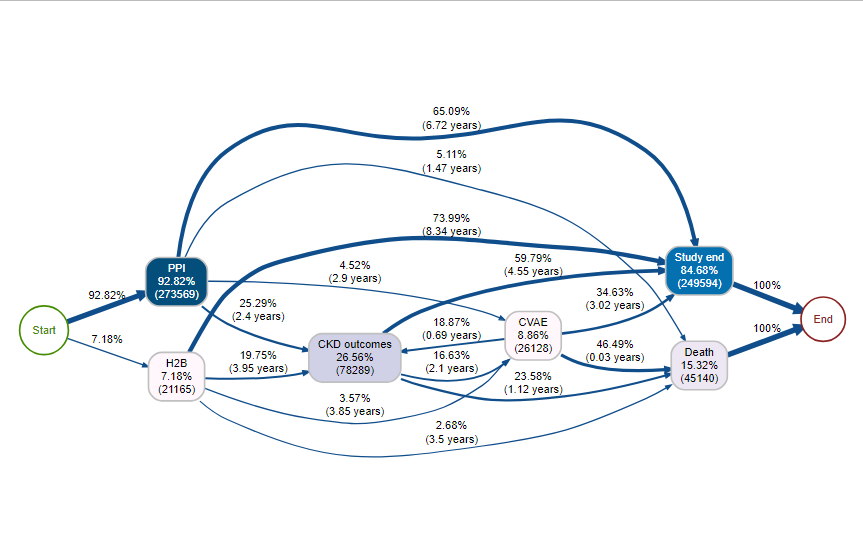

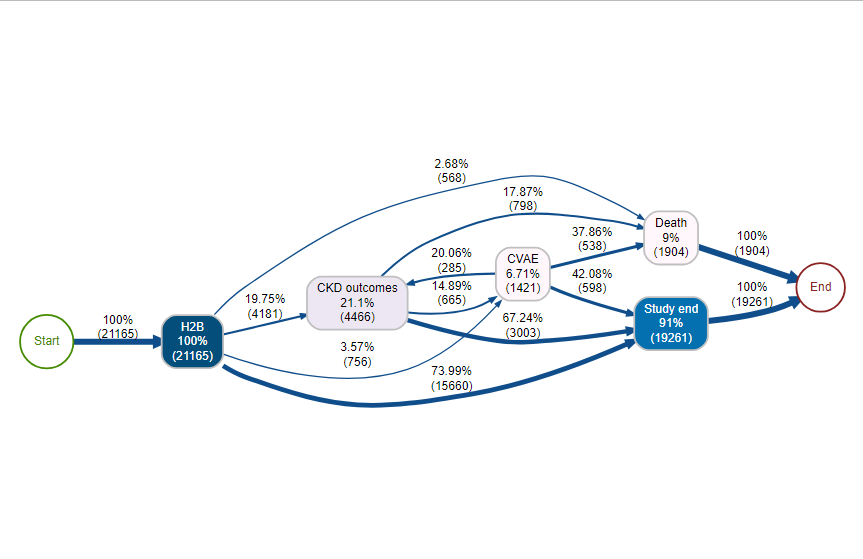

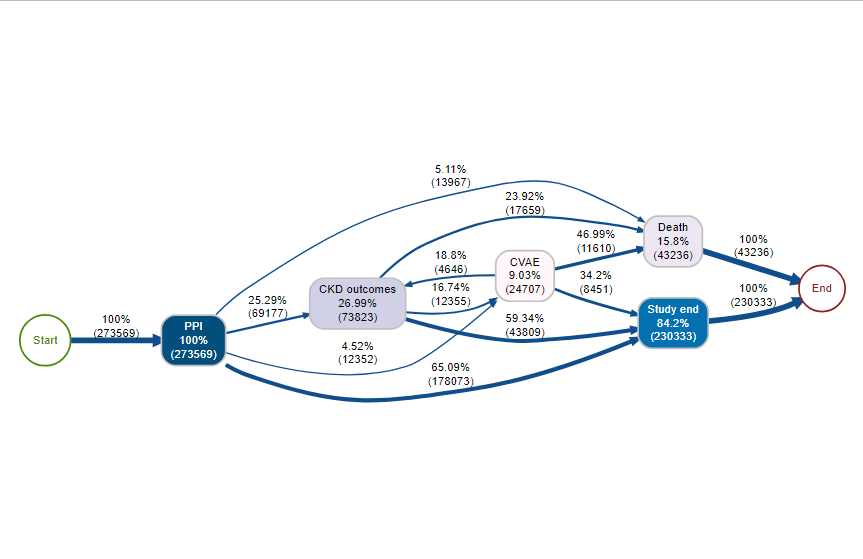


### Supplemental Table 2. Summary of three competing risk models evaluating associations with cardiovascular adverse events (CVAE) and chronic kidney disease (CKD). Model 1: medication exposure (PPI vs. H₂B) in relation to CVAE. Model 2: medication exposure (PPI vs. H₂B) in relation to CKD. Model 3: CKD status (Yes vs. No) in relation to CVAE, adjusted for medication exposure. Results are reported as subdistribution hazard ratios (SHR) with 95% confidence intervals (CI) and p-values

|  | Medication (PPI vs H2B) → CVAE | | Medication (PPI vs H2B) → CKD | | CKD (Yes vs No) → CVAE | |
| --- | --- | --- | --- | --- | --- | --- |
| **Variables** | **SHR** | **P-value** | **SHR** | **P-value** | **SHR** | **P-value** |
| Group |  |  |  |  |  |  |
| H2B | Reference | Reference | Reference | Reference | Reference | Reference |
| PPI | 0.97 (0.92, 1.02) | 0.307 | 1.10 (1.07, 1.13) | <0.001 | 1.10 (1.04, 1.17) | 0.002 |
| Age | 1.07 (1.06, 1.07) | <0.001 | 1.02 (1.02, 1.03) | <0.001 | 1.07 (1.07, 1.07) | <0.001 |
| Baseline eGFR | 1.00 (1.00, 1.00) | 0.001 | 0.97 (0.97, 0.97) | <0.001 | 1.01 (1.00, 1.01) | <0.001 |
| Gender |  |  |  |  |  |  |
| Male | Reference | Reference | Reference | Reference | Reference | Reference |
| Female | 0.69 (0.67, 0.71) | <0.001 | 0.95 (0.93, 0.96) | <0.001 | 0.66 (0.64, 0.68) | <0.001 |
| Gastrointestinal diseases |  |  |  |  |  |  |
| No | Reference | Reference | Reference | Reference | Reference | Reference |
| Yes | 1.11 (1.07, 1.15) | <0.001 | 1.12 (1.10, 1.15) | <0.001 | 1.09 (1.04, 1.13) | <0.001 |
| Peripheral vascular diseases |  |  |  |  |  |  |
| No | Reference | Reference | Reference | Reference | Reference | Reference |
| Yes | 1.38 (1.31, 1.46) | <0.001 | 1.34 (1.29, 1.40) | <0.001 | 1.39 (1.30, 1.50) | <0.001 |
| Hypertension |  |  |  |  |  |  |
| No | Reference | Reference | Reference | Reference | Reference | Reference |
| Yes | 1.48 (1.43, 1.52) | <0.001 | 1.45 (1.42, 1.47) | <0.001 | 1.39 (1.34, 1.44) | <0.001 |
| Diabetes mellitus |  |  |  |  |  |  |
| No | Reference | Reference | Reference | Reference | Reference | Reference |
| Yes | 1.36 (1.32, 1.41) | <0.001 | 1.39 (1.36, 1.42) | <0.001 | 1.38 (1.33, 1.44) | <0.001 |
| COPD |  |  |  |  |  |  |
| No | Reference | Reference | Reference | Reference | Reference | Reference |
| Yes | 1.33 (1.29, 1.37) | <0.001 | 1.13 (1.11, 1.15) | <0.001 | 1.37 (1.32, 1.43) | <0.001 |
| Hyperlipidemia |  |  |  |  |  |  |
| No | Reference | Reference | Reference | Reference | Reference | Reference |
| Yes | 1.00 (0.95, 1.04) | 0.877 | 1.05 (1.02, 1.08) | <0.001 | 1.10 (1.04, 1.16) | <0.001 |
| NSAIDs/Aspirin |  |  |  |  |  |  |
| No | Reference | Reference | Reference | Reference | Reference | Reference |
| Yes | 0.88 (0.86, 0.91) | <0.001 | 0.93 (0.92, 0.95) | <0.001 | 1.03 (1.00, 1.06) | 0.059 |
| Statins |  |  |  |  |  |  |
| No | Reference | Reference | Reference | Reference | Reference | Reference |
| Yes | 0.95 (0.92, 0.98) | <0.001 | 0.98 (0.96, 1.00) | 0.092 | 0.95 (0.92, 0.99) | 0.013 |
| Antithrombotics |  |  |  |  |  |  |
| No | Reference | Reference | Reference | Reference | Reference | Reference |
| Yes | 1.25 (1.21, 1.29) | <0.001 | 1.15 (1.13, 1.17) | <0.001 | 1.23 (1.19, 1.27) | <0.001 |
| SSRIs |  |  |  |  |  |  |
| No | Reference | Reference | Reference | Reference | Reference | Reference |
| Yes | 1.04 (1.01, 1.08) | 0.024 | 1.02 (1.00, 1.03) | 0.069 | 1.10 (1.05, 1.15) | <0.001 |
| Smoking |  |  |  |  |  |  |
| No | Reference | Reference | Reference | Reference | Reference | Reference |
| Yes | 1.37 (1.23, 1.54) | <0.001 | 1.18 (1.09, 1.27) | <0.001 | 1.63 (1.41, 1.89) | <0.001 |
| CKD |  |  |  |  |  |  |
| No | NA | NA | NA | NA | Reference | Reference |
| Yes | NA | NA | NA | NA | 1.34 (1.29, 1.39) | <0.001 |

### Supplemental Table 3. ICD-10 and ATC code for identification of comorbidities, and medications

| **Variables** | **Definition** |
| --- | --- |
| **Kidney function** | **eGFR (CKD-EPI equation)** |
| **Comorbidities** | **ICD-10 codes/ATC codes** |
| Gastroesophageal reflux disease | K21 |
| Upper gastrointestinal tract bleeding | K922 |
| Ulcer disease | K221, K25, K26, K27, K28 |
| H. Pylori infection | B980 |
| Peripheral vascular disease | I70, I71, I731, I738, I739, I771, I790, I792, K551, K558, K559, Z958, Z959 |
| Hypertension | I10-15; **ATC:** C03, C07, C08, C09 |
| Diabetes mellitus | E10-14; **ATC:** A10A, A10B |
| Chronic obstructive pulmonary disease | I278, I279, J40-47, J60-67, J684, J701, J703 |
| Dyslipidaemia | E780, E781, E782, E784, E785 |
| Smoking | Z720, F172 |
| **Medications** | **ATC codes** |
| Proton pump inhibitors | A02BC |
| H2 blockers | A02BA |
| NSAID/aspirin | M01A |
| Statin | C10AA |
| Antithrombotic | B01A |
| SSRI | N06AB |


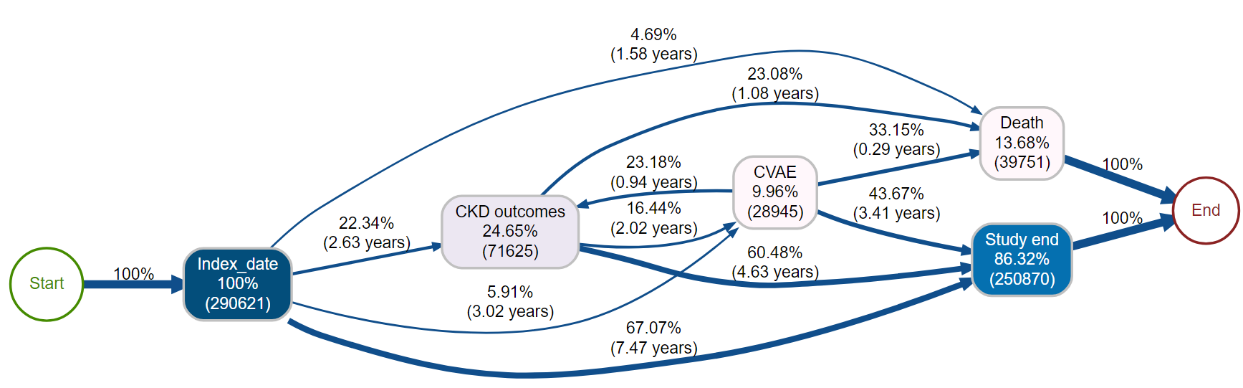

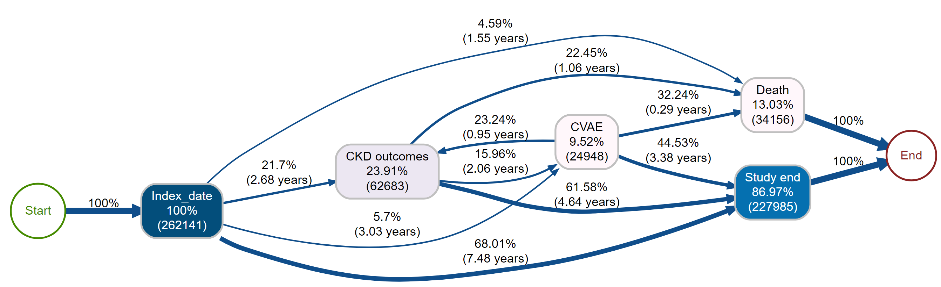

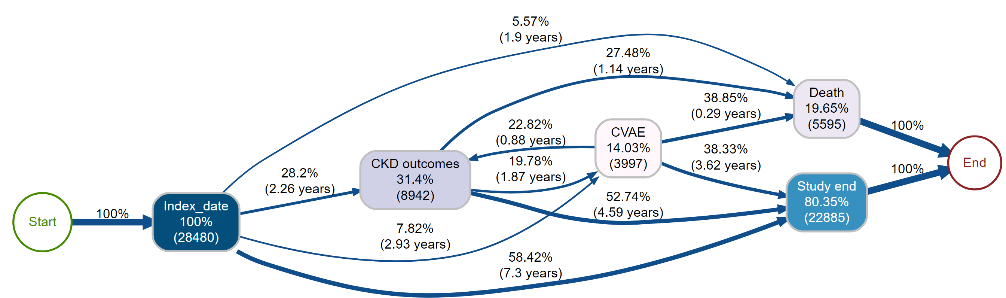

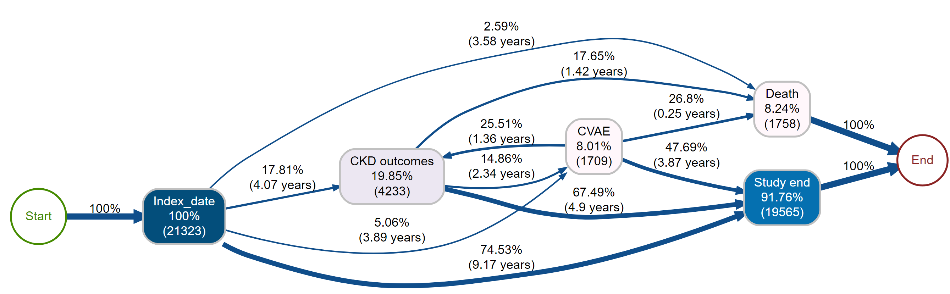

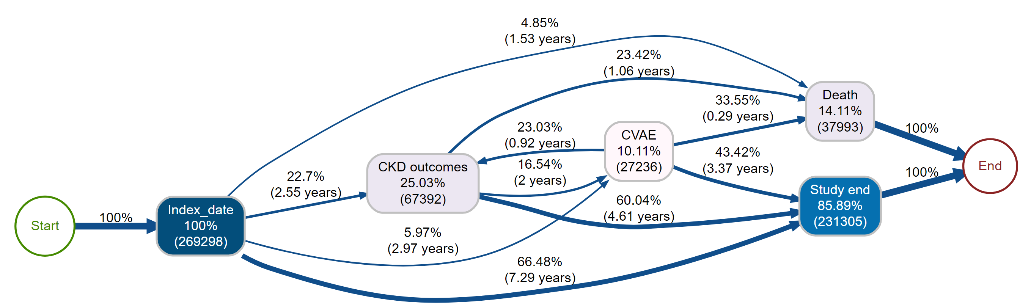


Filter by Drug Group: PPI vs. H2B

**Filter**: Gastrointestinal diseases vs. Non-gastrointestinal diseases

Overall


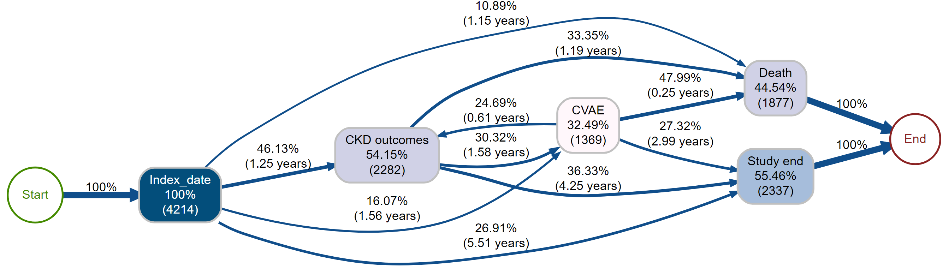

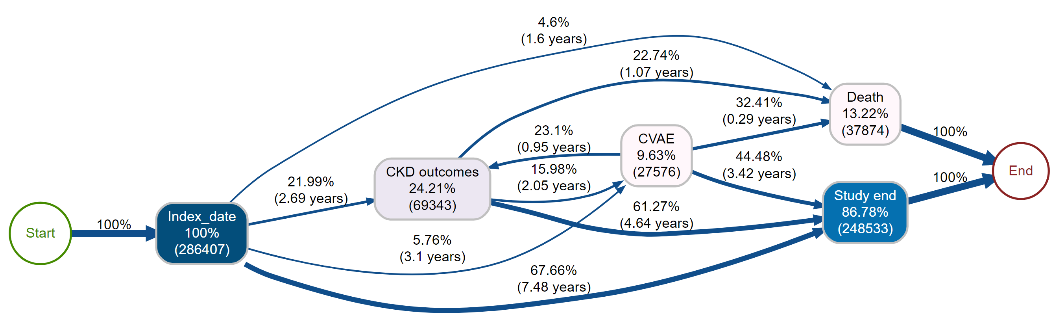

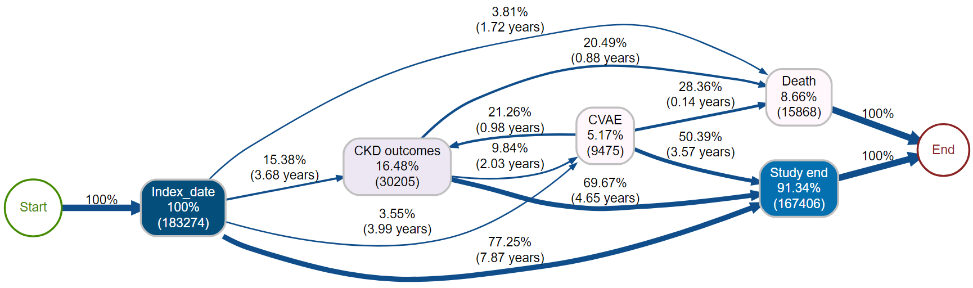

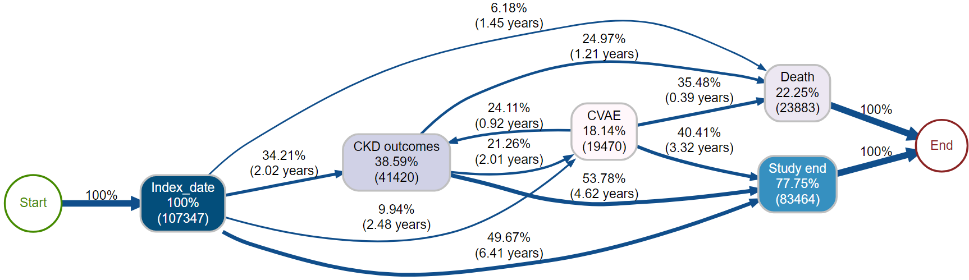


**Filter**: Peripheral vascular disease vs. Non**-peripheral vascular**

**Filter**: Hypertension vs. Non**-hypertension**

**Filter**: Diabetes vs. Non-diabetes


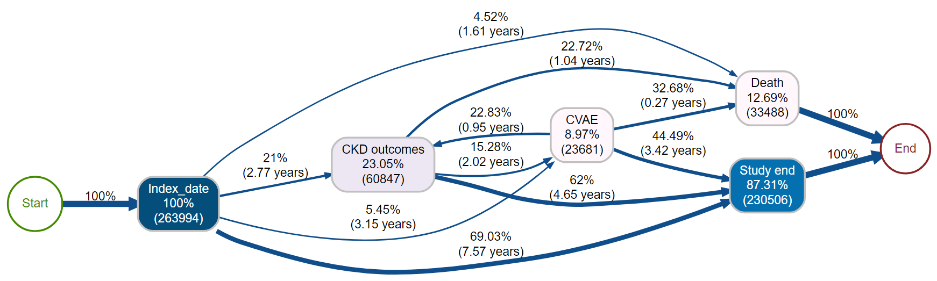

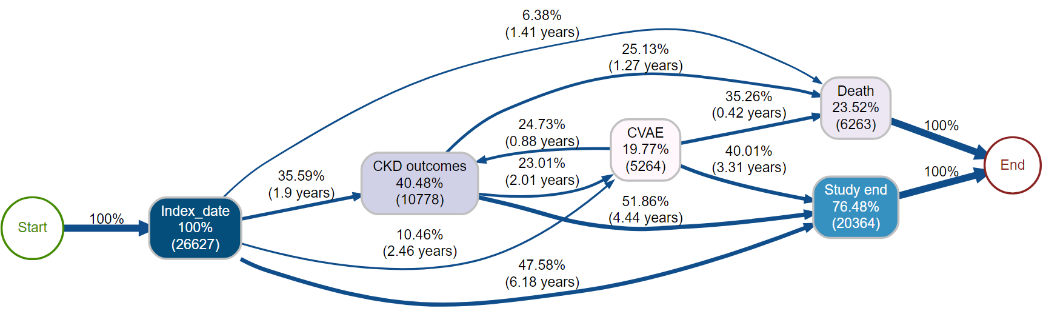

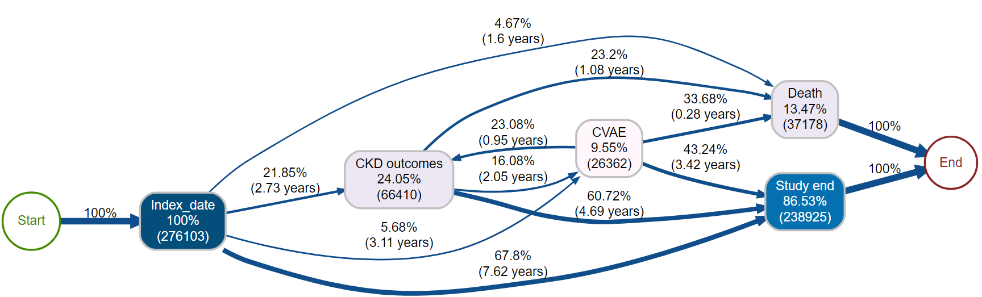

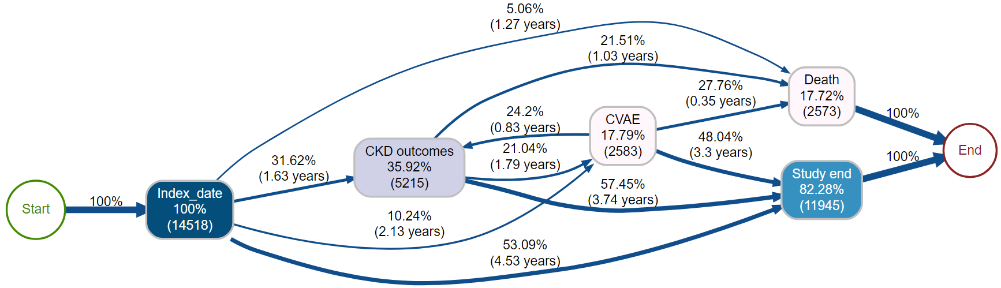


**Filter**: COPD vs. Non-COPD


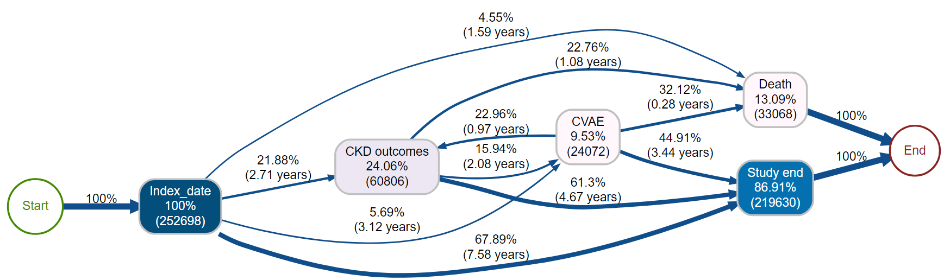

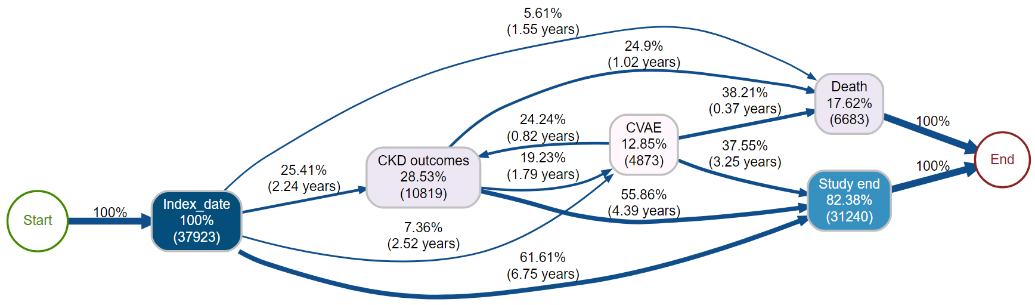


**Filter**: Dyslipidemia vs. Non-dyslipidemia

**Filter**: Statins vs. Non-statins


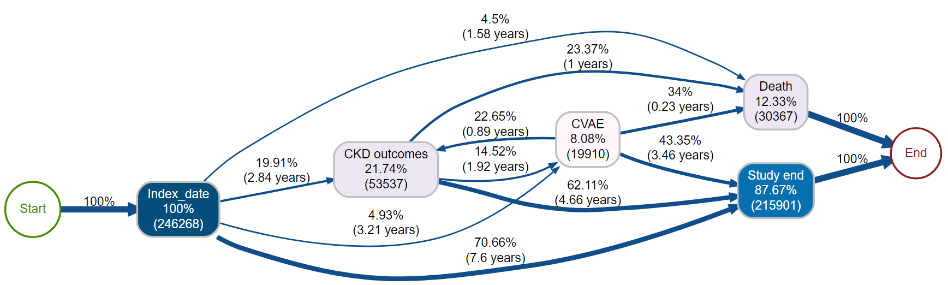

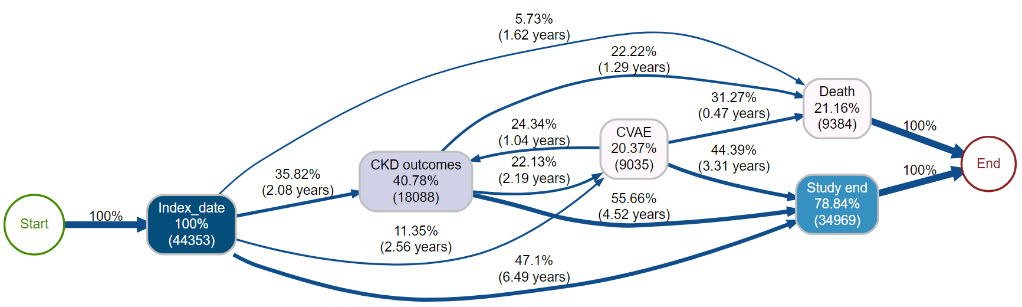

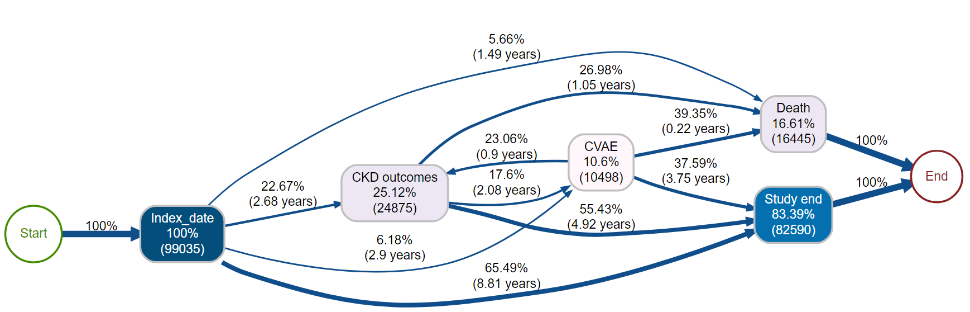

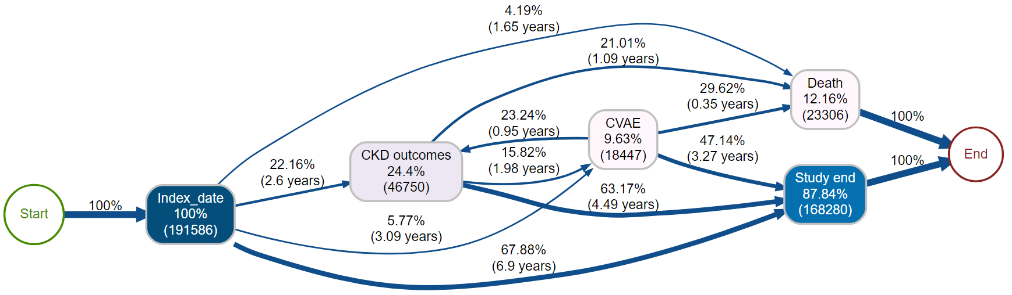

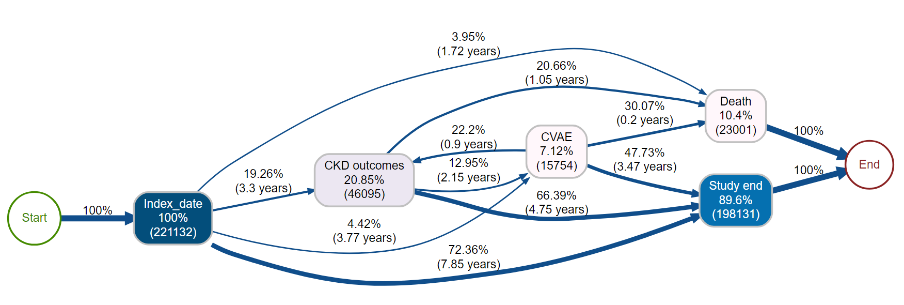

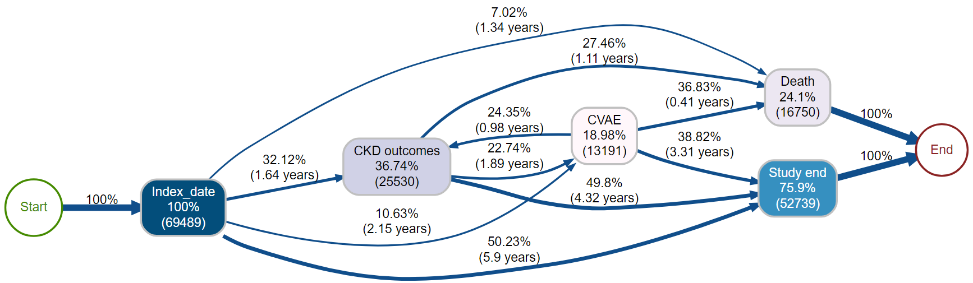


**Filter**: NSAIDS vs. Non-NSAIDS

**Filter**: Antithrombotics vs. Non-antithrombotics

**Filter**: SSRIs vs. Non**-**SSRIs


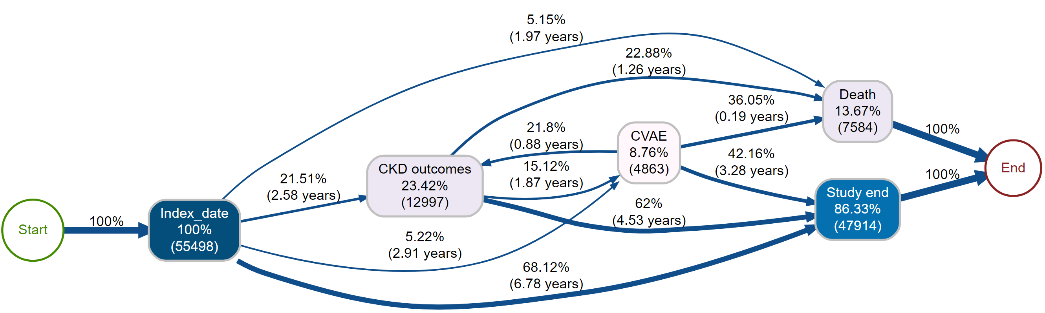

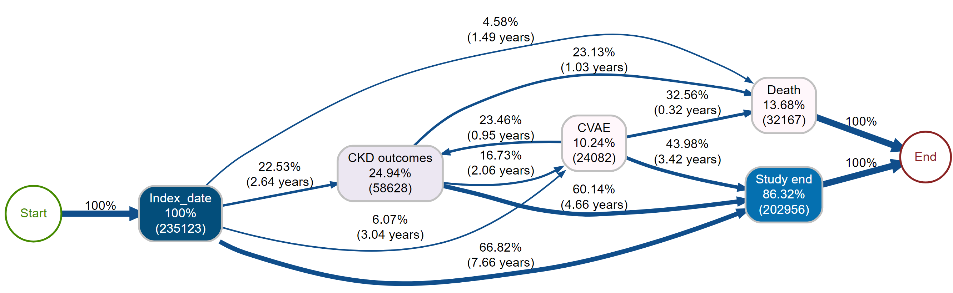


### Supplemental Figure 2. Interactive process maps; These maps display the entire cohort and progressively filter individuals based on baseline profiles. The nodes are the predefined events with a darker colour, which means more event frequency; the edge (arrows) represent time-ordered sequences of traces; the thickness of these arrows corresponds to the frequency of occurrence. The definition of each node is: "Index date" - start date for Proton Pump Inhibitors (PPI) or H2 Blockers (H2B); "CKD outcomes" – a composite outcome including eGFR decline 30% from baseline, CKD incidence, KRT and death due to CKD; "CVAE" – a composite outcome including Myocardial Infarction, Heart Failure, Stroke and Death due to Cardiovascular Events; “Death”- all-cause mortality; “Study end” -the date on which follow-up ended for participants who did not experience any events. Each node displays two statistics: Relative Case Frequency: The proportion of cases in which the event was executed. Absolute-Case Frequency (in brackets): The total number of cases undergoing the event. Each edge displays two additional statistics: Relative-Antecedent Frequency, which is the proportion of source (antecedent) cases directly followed by the target cases. Median Time: The median duration from the antecedent event to the target event.
